# Progression of skin lesions in Warburg-Cinotti syndrome

**DOI:** 10.1016/j.jdcr.2021.12.006

**Published:** 2021-12-15

**Authors:** Christopher A. Ours, Leslie G. Biesecker, Thomas N. Darling

**Affiliations:** a Center for Precision Health Research, National Human Genome Research Institute, National Institutes of Health, Bethesda, Maryland; b Department of Dermatology, Uniformed Services University, Bethesda, Maryland

**Keywords:** contracture, *DDR2*, keloid, rare disease, Warburg-Cinotti Syndrome, DDR2, discoidin domain receptor 2, WCS, Warburg-Cinotti syndrome

## Introduction

Warburg-Cinotti syndrome (WCS) is a rare condition inherited in an autosomal dominant pattern caused by activating variants in the discoidin domain receptor 2 (*DDR2*) gene.^1^ Manifestations include corneal vascularization and pannus, contractures of the hand, acro-osteolysis, and multiple dermatologic findings. The skin can be affected by chronic ulcerations, pseudosyndactyly of the toes, hyperkeratotic and verrucoid lesions, cicatricial alopecia, and spontaneous polymorphic plaques having features similar to keloids. At the time of this report, only 6 individuals, including the patient presented in this report, had been described as having WCS. This report demonstrates the progressive nature of skin lesions in an adult with WCS.

## Case report

**Fig 1:**
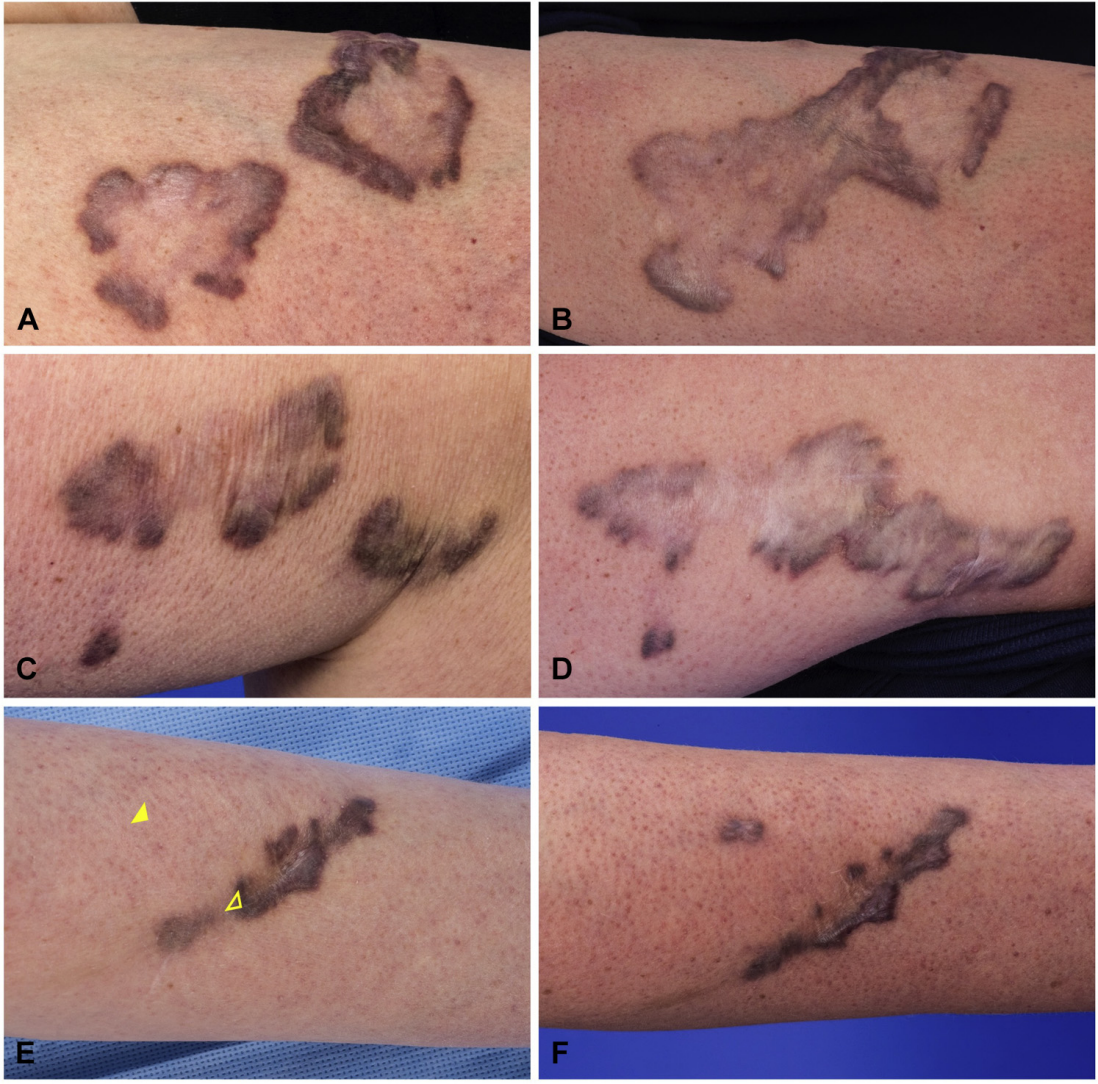
Progression of skin lesions at age 31.5 years (**A, C, E**) and 34 years (**B, D, F**). **A** and **B,** Right upper arm. **C** and **D,** Left upper arm. **E** and **F,** Skin biopsy sites of unaffected (*closed arrowhead*) and affected (*open arrowhead*) skin on the left forearm.

A 34-year-old woman with WCS was seen at a 30-month interval follow-up as part of a rare disease natural history study (10-HG-0065). The original presentation and identification of the *DDR2* variant c.2219A>G p.(Tyr740Cys) in this patient was included in a prior publication in which the patient was identified as “Individual 3.”^1^ Her manifestations of WCS began at the age of 5 years with the appearance of a painless, nonpruritic, dark purple papule on her left forearm that remained unchanged for several years. During early adolescence, a second similar lesion developed on her left upper arm. This lesion increased in size and was treated with intralesional steroid injection followed by excision and laser treatments. The clinical record of this treatment and the histopathology of this lesion are unavailable, but the lesion did not recur. During her second and third decades of life, multiple reddish-brown plaques developed, with the largest on her upper arms. These firm, polymorphic plaques presented with polygonal, annular, polycyclic, and linear shapes and have increased in pigmentation, extended circumferentially, and coalesced with nearby lesions (Fig 1, *A* to *D*). She did not seek further treatment of these lesions. There is no known history of preceding trauma, insect bite, scratch, or inflammation in most areas where these lesions developed.

**Fig 2:**
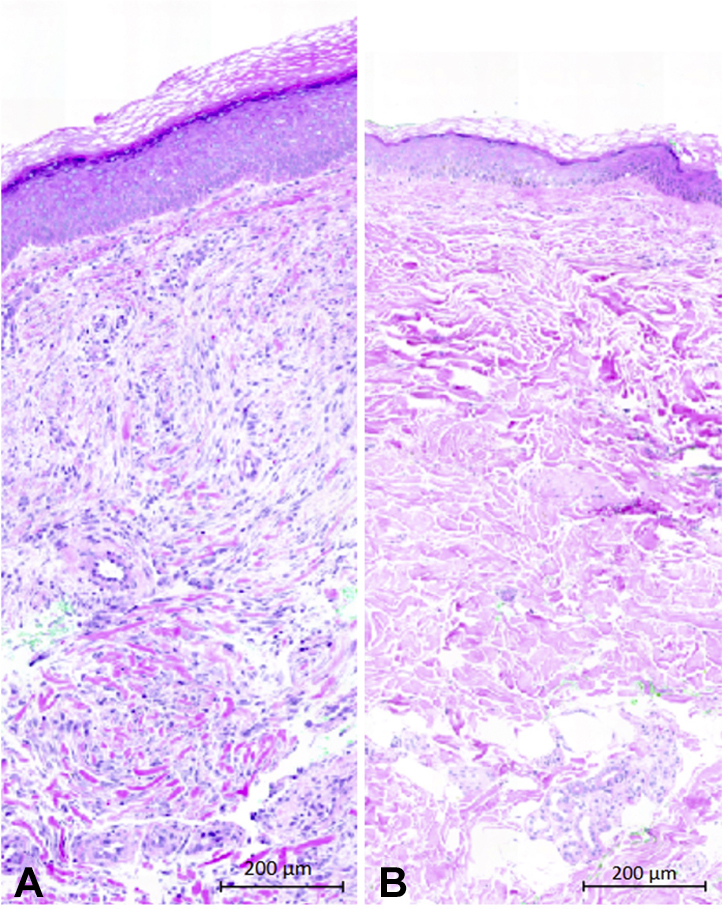
Hematoxylin and eosin-stained sections from (**A**) affected skin demonstrating a thick flattened epidermis, increased cellularity in the dermis, and foci of thickened collagen fibers in the dermis, and (**B**) unaffected skin.

As part of our study, biopsies were performed on affected and unaffected skin (Fig 1, *E* and *F*). Histopathologic examination of the lesion showed a thickened flat epidermis, increased vasculature, and fibroblast hyperplasia with formation of coarse collagen fibers involving the whole thickness of the dermis (Fig 2, *A* and *B*). These findings are similar to those previously described.^2^ The unaffected area showed no histologic pathology. A small, pigmented papule developed at the site of the unaffected skin biopsy that has not increased in size since its appearance.

**Fig 3:**
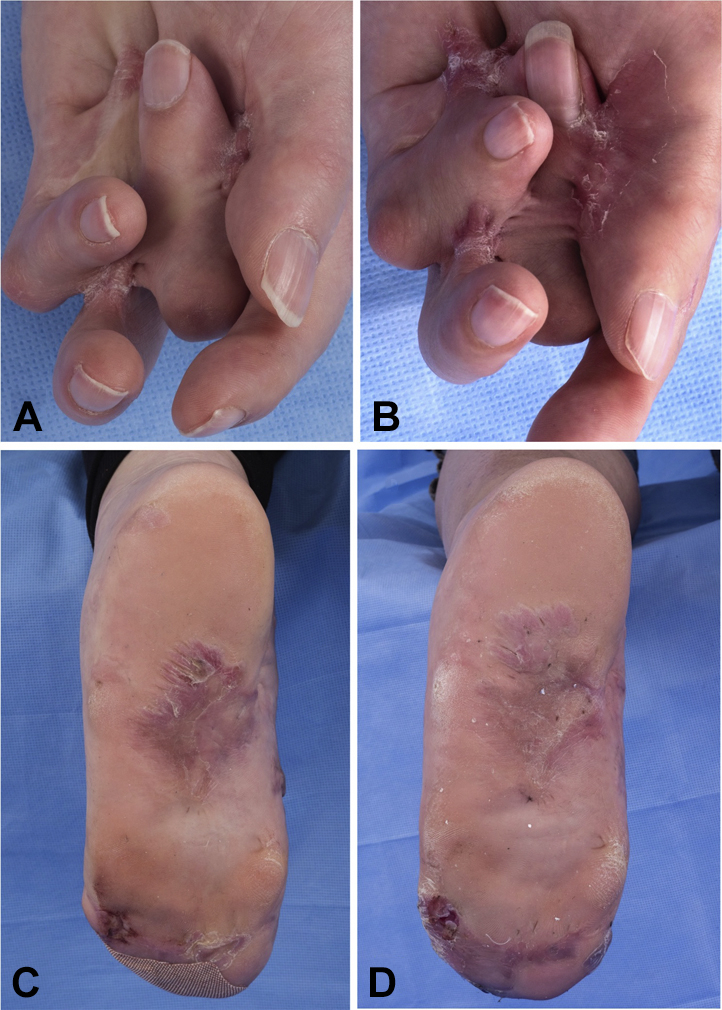
Progression of hand contractures and cutaneous adhesions at age 31.5 years (**A, C**) and 34 years (**B, D**). **A** and **B,** Digital flexion contracture and increasing interdigital and palm-to-digit adhesions of the left hand. **C** and **D,** Pseudosyndactyly of the left foot with chronic ulceration at the base of the fifth digit.

In addition to the plaques, beginning in late adolescence, she has had spontaneous recurrent sterile ulcers of the hands and feet and progressive hand contractures. She underwent a skin graft to a chronic ulcer that healed without hypertrophic scarring. The contractures, which were originally diagnosed as Dupuytren's contracture, did not improve with needle aponeurotomy or collagenase injection. Interdigital and palm-to-digit cutaneous adhesions formed at areas brought in proximity as a result of the contractures and at the site of ulcerations. In a similar manner, bilateral pseudosyndactyly of the toes developed over several years (Fig 3, *A* to *D*).

Extradermatologic features include acro-osteolysis, hypothyroidism, and right eye blindness due to corneal pannus and conjunctival scaring. She has 2 affected children with early hand contractures who have not developed similar skin lesions.

## Discussion

We present this patient to increase awareness of the presentation and progression of WCS. The first signs of WCS may occur in childhood as mild dermatologic findings and are progressive through adulthood. Although they were not present in this patient, additional reported skin findings include decreased subcutaneous tissue, hyperkeratotic and verrucoid lesions, telangiectasias, cicatricial alopecia, and dystrophic nails.^2^^,^^3^

Two variants of *DDR2* have been described as the cause of WCS, c.1829T>C p.(Leu610Pro) and c.2219A>G p.(Tyr740Cys). DDR2 is a fibrillar collagen tyrosine kinase receptor that promotes dermal myofibroblast proliferation, collagen I secretion, and matrix metalloproteinase 2 production.^4^^,^^5^ The variants found in patients affected with WCS result in ligand-independent kinase activity. In contrast to WCS, the loss of function variants in *DDR2* is associated with spondylometaepiphyseal dysplasia and compromised long bone growth.^6^

There is currently no effective therapy for WCS. This patient responded minimally to intralesional steroid injection, but had a single resected lesion that did not recur. Attempts to release the hand contractures have not been effective. Application of dasatinib to patient-derived fibroblasts decreases DDR2 autophosphorylation.^1^ This requires further consideration as a therapeutic agent for WCS. The recognition and diagnosis of WCS is the first step toward understanding this rare condition.

## Conflicts of interest

Dr Biesecker is a member of the Illumina Corp Medical Ethics Board, has received in-kind research support from ArQule, Inc (now wholly owned by 10.13039/100004334Merck, Inc), and receives honoraria from Cold Spring Harbor Press. Drs Ours and Darling have no conflicts of interest to declare.
